# Temporary transvenous external pacing for cardiac MRI in a pediatric patient

**DOI:** 10.1007/s00247-025-06325-z

**Published:** 2025-07-31

**Authors:** Mehrad Rokni, Shotaro Naganawa, Misagh Piran, Daniel Cortez, Ahmadreza Ghasemiesfe

**Affiliations:** 1 University of California Davis, 4860 Y St, Suite 3100 Sacramento, 95817 USA; 2 UC Davis Health System, Sacramento, USA

**Keywords:** Arrhythmias, cardiac, Cardiac pacing, artificial, Heart block, Magnetic resonance imaging, Pacemaker, artificial, Pediatrics

## Abstract

This report discusses the use of temporary transvenous external cardiac pacing during cardiac magnetic resonance imaging (MRI). Despite its feasibility having been explored in adults, its application in pediatric patients is not well established. We present a case of a 6-year-old female with a high-degree atrioventricular (AV) block managed with an externalized temporary pacing system for MRI. This system enabled cardiac MRI for further treatment planning, eliminating the immediate need for permanent pacemaker implantation. This case underscores the feasibility of conducting cardiac MRI in pediatric patients requiring temporary pacing, contingent upon the utilization of appropriate equipment and protocols.

## Introduction

Cardiac magnetic resonance imaging (MRI) is a vital tool for diagnosing pediatric cardiac conditions, providing detailed tissue characterization and quantitative functional analysis without the ionizing radiation harmful to children [1–5]. Temporary transvenous pacing stabilizes heart rhythm in patients with unstable arrhythmias, but its use during MRI is complex due to potential interactions between the magnetic field and pacing devices [2, 4]. This technique has been documented in adults, with a 44-patient series demonstrating its feasibility and safety under strict protocols [2]. Its use in pediatric literature is limited, potentially due to fewer pediatric patients requiring MRI during temporary pacing, although technical and safety concerns related to smaller anatomies and limited pediatric-specific data may also contribute [1, 3]. This report illustrates the integration of temporary externalized pacing with MRI for pediatric cardiac patients, offering a novel approach to diagnostic imaging in this population [6, 7].

## Case report

**Fig. 1:**
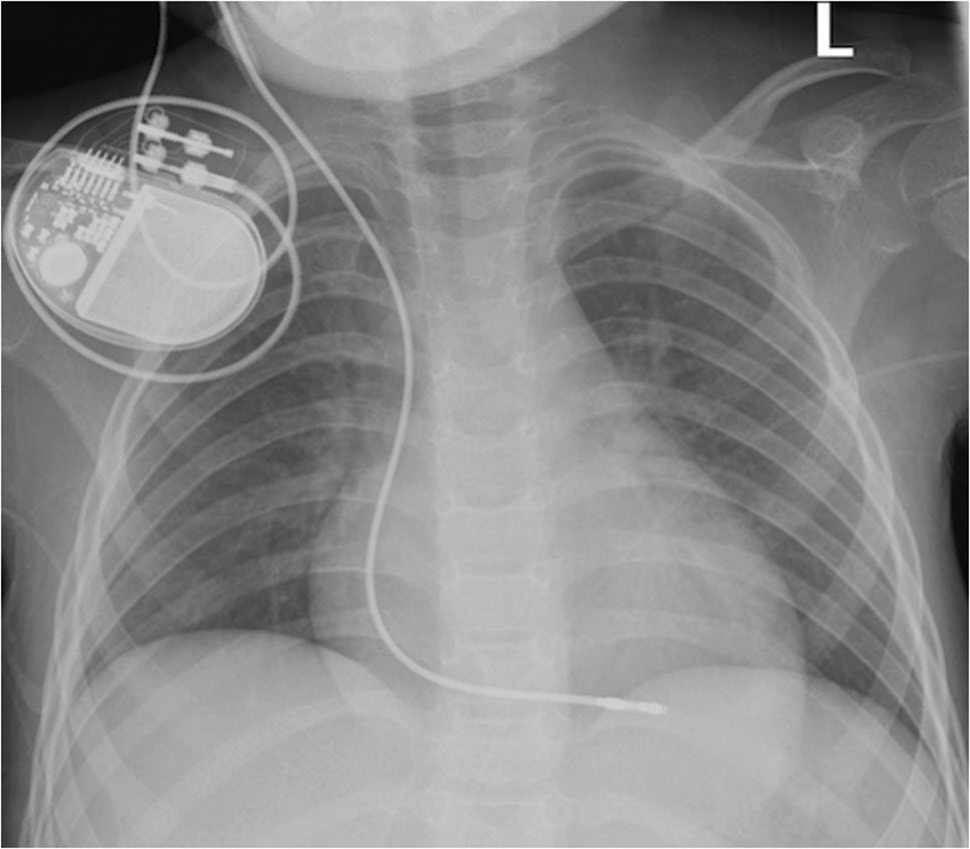
A 6-year-old girl presented with bradycardia secondary to type II Mobitz and intermittent complete heart block. Anteroposterior chest radiograph shows the appropriate placement of the transvenous pacing lead, with its tip in the right ventricle and the externalized pacemaker generator taped to the skin

**Fig. 2:**
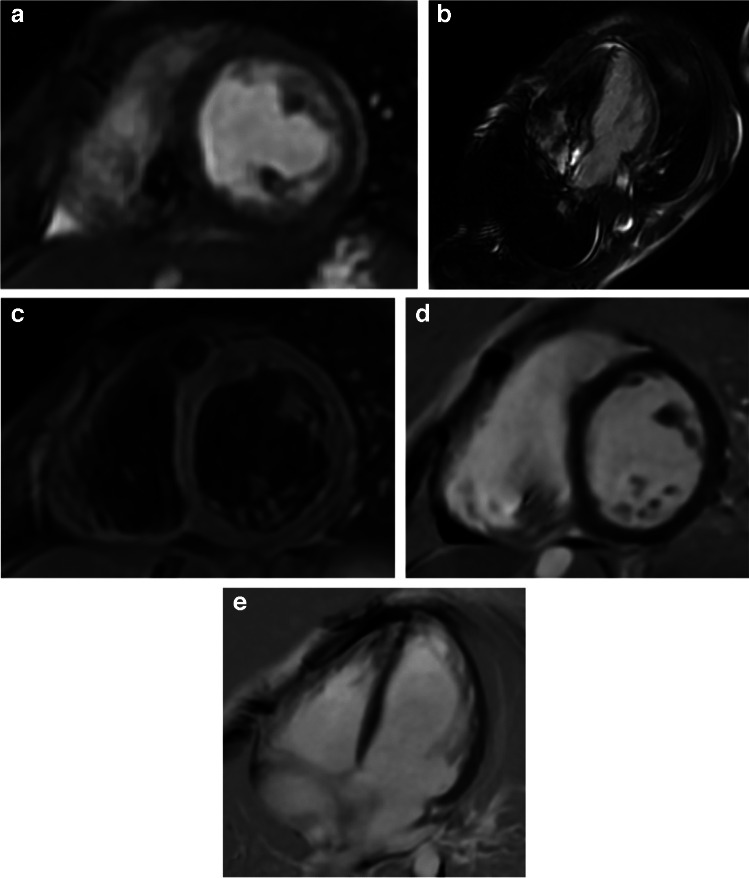
A 6-year-old girl with high-degree atrioventricular (AV) heart block. **a** Short-axis, unenhanced, balanced steady-state free precession (bSSFP) cardiac MR image shows acceptable image quality for evaluating cardiac motion and myocardial structure with minimal artifact. **b** Four-chamber unenhanced bSSFP cardiac MR image demonstrates susceptibility artifact in the right chest wall related to the externalized pacemaker generator. Susceptibility artifacts are also observed in the right cardiac chambers due to the pacing lead. These artifacts affected <5% of the imaging field and did not impact functional analysis. **c** Short-axis unenhanced triple inversion recovery T2-weighted cardiac MR image shows no myocardial edema and no significant artifact. **d** Short-axis fast gradient echo (FGRE) late gadolinium enhancement (LGE) cardiac MR image shows no abnormal myocardial enhancement. **e** Four-chamber FGRE late gadolinium enhancement (LGE) cardiac MR image shows no abnormal myocardial enhancement

This case was deemed exempt by the Institutional Review Board (IRB) and parental consent was obtained for anonymized data publication. A 6-year-old female presented with bradycardia secondary to type II Mobitz and intermittent complete heart block. Initial evaluation included laboratory tests that showed elevated inflammatory markers (CRP 5.6 mg/L, ESR 63 mm/h) and troponin T levels (peaking at 39 ng/L on day 3). Mild metabolic acidosis was evident (total CO_2_ of 14 mmol/L). Infectious causes, such as Lyme disease and influenza, which are common etiologies for acute heart block in children, were investigated but ruled out through serologic testing [3]. Initial echocardiography confirmed bradycardia without structural abnormalities. An abdominal ultrasound ruled out systemic causes such as hepatic or renal pathology, but the etiology of the conduction abnormalities remained unclear. A temporary transvenous pacing lead was placed under fluoroscopic guidance to stabilize the patient for advanced imaging (Fig. 1) [6]. Cardiac MRI was performed to evaluate potential structural and inflammatory causes of AV block (see Fig. 2 for representative MRI images) [5]. There was no significant artifact related to the generator or transvenous lead. Imaging ruled out myocarditis and structural defects but identified a mildly reduced left ventricular ejection fraction (LVEF; 45%), suggesting primary conduction system involvement [5]. Her diagnosis remains non-specific but is classified as mitochondrial DNA-deletion syndrome. These findings guided further management, including genetic testing and permanent pacemaker implantation due to persistent heart block [3, 8].

## Technique overview

Parental consent for off-label cardiac device use in the magnetic environment was obtained, addressing risks of heating and malfunction. Temporary pacing was established using a transvenous active-fixation lead (Tendril STS Model 2088 T, 52 cm; Abbott, Chicago, IL, USA) connected to a Dual-Chamber Pacemaker (Assurity MRI™ Dual-Chamber Pacemaker Model PM2272, Abbott, Chicago, IL, USA) externalized and taped to the skin [6]. Gauze separated the pacemaker from the skin to reduce radiofrequency (RF)-induced heating risks. Pre-procedure, the electrophysiology (EP) team programmed the pacemaker to asynchronous ventricular pacing mode (VOO) at 70 beats per minute, with an output of 3.5 V at 0.4 ms, to prevent magnetic interference [2]. The atrial port was plugged per manufacturer MRI specifications to ensure safety [4]. After the MR protocol, specific absorption rate (SAR) was optimized, and institutional protocols for cardiac implantable electronic devices (CIEDs) were followed, involving EP-radiology collaboration. The MRI was conducted in a 1.5-T scanner (MAGNETOM Aera, Siemens Healthineers, Erlangen, Germany) using a 16-channel cardiac coil in a normal MR mode [7]. The imaging protocol included short-axis and four-chamber cine imaging, short-axis triple inversion recovery, T1 and T2 mapping, and late gadolinium enhancement (LGE) sequences, using segmented breath-hold techniques [5]. The patient remained awake and under close monitoring throughout the scan, facilitated by a short imaging protocol [1]. Continuous real-time non-invasive hemodynamic monitoring with electrocardiogram and pulse oximetry was performed. The surface temperature of the generator was monitored using MRI-compatible fiber-optic sensors (FOT-L-SD, FISO Technologies, Quebec, Canada) and no heating was detected [4].

## Discussion

This case demonstrates the feasibility of temporary transvenous pacing with an externalized permanent pacemaker during cardiac MRI in a pediatric patient, aligning with Kakarla et al., who reported safe cardiac MRI in three pediatric myocarditis cases with temporary pacing [1]. Unlike traditional temporary pacemakers (e.g., Model 5392, Medtronic, Minneapolis, MN, USA), which are not MRI-safe due to risks of heating and malfunction, the externalized Assurity MRI™ pacemaker system is MRI-conditional, offering a safer alternative for diagnostic imaging [2, 7]. The externalized Assurity MRI™ pacemaker generator, taped to the skin, and the transvenous lead, positioned in the right ventricle, minimized artifacts compared to traditional pacemakers [2]. Similar to Kakarla et al., our case showed minimal artifacts (<5% of the imaging field), enhancing diagnostic clarity [1]. Cardiac MRI provided critical diagnostic insights, identifying primary conduction system involvement without myocarditis and guiding permanent pacemaker implantation [5, 8].

Compared to echocardiography, which lacks detailed tissue characterization, and computed tomography (CT), which involves ionizing radiation and limited functional assessment, cardiac MRI is superior for pediatric patients requiring repeated imaging [5, 8]. In adults, Fyenbo et al. reported safe MRI with temporary external pacemakers in 44 patients, with minimal artifacts and no adverse events, using VOO mode and strict SAR limits [2]. Our case extends these findings to pediatrics, where smaller anatomies necessitated adjustments, such as precise lead placement and tailored pacing settings, to ensure safety and image quality [3, 6].

Pediatric patients present unique challenges for temporary pacing during MRI, including smaller venous anatomy requiring meticulous lead positioning and higher baseline heart rates necessitating customized pacing parameters [3, 7]. These factors, combined with limited pediatric-specific data on MRI-conditional devices, underscore the need for specialized protocols. Safety measures in our case included VOO pacing, SAR optimization below 2 W/kg, gauze insulation, and continuous real-time monitoring of hemodynamics and surface temperature using fiber-optic sensors, with no RF-induced heating detected [2, 4]. Institutional CIED protocols, involving pre-procedure EP consultations and real-time radiology coordination, ensured rigorous risk management [4, 7]. Unlike Kakarla et al., who used general anesthesia, our patient remained awake, reducing procedural complexity and resource use [1].

However, pediatric-specific data remain limited, and challenges include the need for specialized equipment and multidisciplinary expertise. Future multi-center studies should validate MRI-conditional pacing systems across diverse pediatric populations, while the development of smaller, pediatric-specific devices could further reduce artifacts and enhance safety [6, 7].

## Conclusion

Temporary transvenous external cardiac pacing combined with cardiac MRI offers a powerful diagnostic tool for pediatric patients with severe conduction abnormalities. This approach provides high-quality imaging with minimal artifacts, enabling accurate diagnosis while minimizing risks through rigorous safety protocols. Further research is required to standardize protocols and confirm safety across diverse pediatric populations.

## Data Availability

No datasets were generated or analysed during the current study.
